# Understanding Clinical Effectiveness and Safety Implications of Botulinum Toxin in Children: A Narrative Review of the Literature

**DOI:** 10.3390/toxins16070306

**Published:** 2024-07-04

**Authors:** Salvatore Crisafulli, Francesco Ciccimarra, Zakir Khan, Francesco Maccarrone, Gianluca Trifirò

**Affiliations:** 1 Department of Medicine, University of Verona, 37124 Verona, Italy; salvatore.crisafulli@univr.it; 2 Department of Diagnostics and Public Health, University of Verona, 37124 Verona, Italy; francesco.ciccimarra@univr.it (F.C.); francesco.maccarrone@univr.it (F.M.); 3 Department of Pharmacy Practice, Riphah Institute of Pharmaceutical Sciences, Riphah International University, Gulberg Green Campus, Islamabad 44000, Pakistan; zakir.khan@riphah.edu.pk

**Keywords:** botulinum toxin, pediatrics, clinical safety, clinical effectiveness

## Abstract

Since its first approval by the Food and Drug Administration in 1989 for strabismus, botulinum toxin indications of use have been widely expanded. Due to its anticholinergic properties, this toxin is currently approved in adult patients for the treatment of a wide range of neuromuscular, otolaryngologic, orthopedic, gastrointestinal, and urologic disorders. Approved pediatric indications of use include the treatment of blepharospasm associated with dystonia, strabismus, lower-limb spasticity, focal spasticity in patients with cerebral palsy, and neurogenic detrusor overactivity. Alongside these approved indications, botulinum toxin is extensively used off-label. Although several clinical studies have shown that botulinum toxin is effective and well-tolerated in children, uncertainties persist regarding its long-term effects on growth and appropriate dosing in this population. As such, further research is needed to better define the botulinum toxin risk–benefit profile and expand approved uses in pediatrics. This narrative review aimed to provide a broad overview of the evidence concerning the clinical effectiveness and safety of BoNT with respect to its principal authorized and non-authorized pediatric therapeutic indications, as well as to describe perspectives on its future use in children.

## 1. Introduction

Botulinum neurotoxin (BoNT) is an extremely poisonous toxin secreted by the *Clostridium botulinum*, an anaerobic, Gram-positive, rod-shaped bacterium [1]. The term “botulinum” is derived from the Latin word “*botulus*”, which means sausage, because the toxin was initially associated with contaminated sausages [2]. To date, based on their structure, seven serotypes have been identified, labeled as type A through G (i.e., BoNT/A-G), including a total of 40 subtypes [3]. Of these, types A and B have the longest in vivo duration of action (weeks to months) and are commonly used for therapeutic and cosmetic purposes. All BoNT serotypes have a similar structure, consisting of a light chain (LC) weighting about 50 kDa and a heavy chain HC of about 100 kDa. The latter presents an N-terminal translocation domain (HN) and a C-terminal receptor-binding domain (HC). All BoNT subtypes are able to bind gangliosides and synaptic vesicle proteins (e.g., synaptotagmin and synaptic vesicle glycoprotein 2) on the presynaptic membrane [4,5,6]. This binding is followed by internalization into the cell and subsequently into the synaptic vesicle. The binding to the vesicular membrane induces a conformational change in the HN domain, which, in turn, cleaves the LC subunit that translocates to the cytosol. This subunit is a metalloprotease able to cut the bond between the VAMP/synaptobrevin and synaptosomal-associated protein (SNAP)-25 proteins, which are fundamental components for neurotransmitter release, thereby inhibiting the release of acetylcholine from the synaptic terminal [7]. This results in an irreversible presynaptic block of peripheral cholinergic transmission, both at the neuromuscular junction and at sympathetic and parasympathetic terminals. The chemical denervation produced causes the typical weakening effect on muscle activity and the reduction of glandular secretions [1]. Due to its ability to transiently block the release of acetylcholine from the presynaptic terminal of motor nerves, BoNT has been extensively employed for the treatment of several diseases over time. The detailed mechanisms of BoNT action underscore the complex interplay between toxin structure and neuronal function, revealing ongoing research gaps in understanding its full pharmacological potential and clinical implications.

BoNT was first approved by the Food and Drug Administration (FDA) in 1989 for the treatment of strabismus [8], and in 1994 by the European Medicines Agency (EMA) for the treatment of neuromuscular disorders [9]. Over the years, BoNT has been extensively used for the treatment of muscle-hyperactive diseases such as spasticity, dystonia, hemifacial spasms, blepharospasm, tics, tremors, and gastrointestinal tract mobility disorders, as well as other conditions encompassing but not limited to strabismus, hyperhidrosis, chronic migraine, and alopecia areata [10]. However, gaps in our knowledge persist regarding the long-term effects of BoNT on neuromuscular function, especially in diverse patient populations and across varying disease severities.

To date, four different BoNT-A formulations, namely, onabotulinumtoxinA (OnaBoNT-A), abobotulinumtoxinA (AboBoNT-A), incobotulinumtoxinA (IncoBoNT-A), and prabotulinumtoxinA, and one formulation of BoNT-B (i.e., rimabotulinumtoxinB—RimaBoNT-B) have been approved by the FDA. Of these, only OnaBoNT-A, AboBoNT-A, and IncoBoNT-A have also been approved by the EMA. BoNT approved formulations are not interchangeable due to differences in clinical effectiveness, duration, dosing, and immunogenicity [11,12]. These distinctions underscore the ongoing clinical challenge in achieving consistent therapeutic outcomes across various BoNT applications. Currently authorized pediatric therapeutic applications of BoNT include the treatment of (i) blepharospasm associated with dystonia in patients aged ≥12 years; (ii) strabismus in patients aged ≥12 years; (iii) lower-limb spasticity in patients ≥2 years; (iv) focal spasticity in ambulatory patients aged ≥2 years with cerebral palsy (CP); (v) chronic sialorrhea in patients aged ≥2 years; and (vi) neurogenic detrusor overactivity (NDO) in patients aged ≥5 years with an inadequate response or intolerance to anticholinergic medications. Despite demonstrated efficacy in approved indications, substantial gaps remain in the understanding of BoNT’s long-term safety and effectiveness profiles in pediatric patients, particularly in less common conditions and younger age groups.

Alongside its approved indications, BoNT is extensively used off-label to treat a range of neuromuscular, otolaryngologic, orthopedic, gastrointestinal, and urologic pediatric conditions [13]. However, the lack of standardized rating tools for evaluating treatment outcomes and the variability in dosing and administration protocols contribute to uncertainties surrounding off-label use, necessitating further research to establish comprehensive guidelines and safety parameters.

Furthermore, the appropriate administration and dose adjustment of different BoNT formulations, particularly in the pediatric population, are still debated. Concerning off-label indications specifically, such issues highlight the need to conduct clinical studies aimed to further explore the BoNT risk–benefit profile in children and to confirm its appropriate uses beyond currently approved conditions.

The aim of this narrative review is to provide an overview of the evidence concerning the clinical effectiveness and safety of BoNT in its principal authorized and non-authorized pediatric therapeutic indications, as well as to describe the future perspectives for BoNT use in pediatrics.

## 2. Clinical Effectiveness and Safety of Botulinum Toxin in Different Pediatric Therapeutic Indications

Relevant articles concerning the clinical effectiveness and safety of BoNT in pediatric patients were searched in two bibliographic databases (i.e., PubMed and Google Scholar) from their inception to 5 April 2024. The search strategy included terms related to BoNT (i.e., botulinum toxin, onabotulinumtoxinA, abobotulinumtoxinA, incobotulinumtoxinA, prabotulinumtoxinA, and rimabotulinumtoxinB) and the pediatric population. The same search strategy and timeframe were also applied to find ongoing clinical studies registered in clinicaltrials.gov, a public repository managed by the United States National Library of Medicine. Search strategy was limited to BoNT therapeutic indications reported in the FDA and/or EMA summary of product characteristics (SPCs), irrespective of whether they are approved for children or not. The retrieved studies were independently screened by two authors for the inclusion in the review.

### 2.1. Cerebral Palsy

CP is one of the most common causes of motor disability in children, and it refers to a group of neurological conditions (e.g., spasticity, dyskinesia, ataxia and hypotonia) resulting in brain’s impaired ability to regulate movement and maintain posture due to anomalies in brain development. CP’s main symptoms are caused by a lesion in the central nervous system, influencing muscle tone, balance, strength, and selectivity. Children with increased muscular tone develop secondary problems over time, such as muscle contractures and bony deformities [14]. Spasticity typically results from a persistent reduction in inhibitory suprasegmental inputs, leading to increased activity of the alpha motor neuron [15]. As such, CP can significantly impact many aspects of patients’ daily life, eating and sleeping. Furthermore, CP-related motor disorders are often associated with both cognitive and behavioral disturbances [16,17,18,19,20].

According to a recent systematic epidemiological analysis, the CP birth prevalence is 1.6 per 1000 live births [19], with varying rates between high income and low to middle income countries [21,22,23,24,25].

BoNT-A’s therapeutic effects typically last 3–4 months and, as such, repeated treatments are required. Since multiple injections of BoNT-A can trigger an immune response, injections should be spaced at least three months apart to prevent the development of antibody resistance [26,27].

The reversible effect of BoNT injection could be explained by the development of new nerve endings from the terminal axon within the first days within the first days of treatment [26].

Dosages for individual preparations should be determined independently, following the specific dosing instructions for each product, and based on previous responses and clinical experience. The ideal dosage for each muscle is influenced by several factors such as muscle volume, the level of spasticity, and the extent of the muscle’s involvement in the pathological pattern [14,28]. In children with CP, it is preferable not to use BoNT-A as a standalone treatment. According to the 2009 European Consensus, the approach to the treatment of CP-related disorders has to be multidisciplinary and multimodal, including conservative and surgical strategies if required [29].

The report of the Quality Standards Subcommittee of the American Academy of Neurology and the Practice Committee of the Child Neurology Society, published in 2010, showed that BoNT-A is a safe and effective treatment for localized spasticity. Such evidence was based on almost 150 studies on the use of BoNT-A for reduction of spasticity in children with CP, with the majority of them regarding lower extremity spasticity. Adverse events were reported on in 17 studies; most frequent AEs were fatigue, localized pain, and weakness, and all were transient and not serious [30].

The first randomized controlled trial (RCT) to assess the efficacy of the use of BoNT injections in children affected by CP was published in 1994 by Koman et al. [31], and several studies are still ongoing.

In the last two decades, a large number of RCTs showed the efficacy of this drug for the treatment of CP. In particular, it was demonstrated that combining occupational therapy with BoNT-A injections was effective in enhancing the active range of hand function and movement [32,33] and improving comfort levels for non-ambulant children with CP, as compared to occupational therapy alone [34]. Similarly, repeated cycles of AboBoNT-A were proven to significantly reduce upper-limb spasticity, especially when associated with a home-exercise therapy program [35,36]. Results from the XARA study, a phase III RCT with open-label extension, showed that IncoBoNT-A was also effective in improving spasticity in 350 patients with bilateral CP affecting the upper limbs, aged from 2 to 17 years [37]. Spasticity improvements were higher in patients receiving high doses of IncoBoNT-A (8 U/kg) as compared to low doses (2 U/kg group), and they were sustained over further treatment cycles [37].

The same was observed for OnaBoNT-A in combination with standardized occupational therapy. Findings from a phase III multinational RCT on 235 patients aged between 2 to 17 years showed that this toxin (3 and 6 U/kg) improved the dynamic tone, especially in patients with only one affected upper-limb muscle group (i.e., elbow or wrist) [38]. Conversely, no improvements in the range of motion and tone of wrist and elbow muscles were observed when BoNT-A was combined with physical and occupational therapy in children with spastic hemiplegia [39].

Significant improvements in gait patterns have been demonstrated by several RCTs [31,40,41,42,43,44,45,46,47]. In particular, three RCTs investigated the effects of different doses of BoNT on gait measures in patients with CP, finding that higher doses were more effective in as compared to lower doses [43,44,45]. In addition, two RCTs showed that injecting the gastrocnemius–soleus complex for spastic equinus foot was equally effective when performed once per year as compared to three times per year (i.e., every four months) [46,47].

Furthermore, AboBoNT-A, IncoBoNT-A, and OnaBoNT-A were found to be effective and safe in significantly reducing muscle tone in children with dynamic equinus foot deformity, which is one of the most common foot disorders in children with CP [36], as well as in enhancing muscle tone and motor function for children with lower-limb spasticity associated to CP [28,48,49]. On the contrary, a two-year placebo-controlled trial assessing the efficacy of AboBoNT-A for leg spasticity in CP found no cumulative or persistent benefits from repeated BoNT-A injections [50]. Evidence concerning the efficacy of BoNT for the treatment of CP in children aged <2 years is sparse. To date, available evidence comes from a recently published systematic review [51] that identified only two small RCTs assessing BoNT safety and efficacy in improving motor development in this population [52,53]. In particular, the study conducted by Tedroff et al. was a single-blind RCT comparing the combination of BoNT-A and stretching with stretching alone on a total of 16 children with CP aged between 11 and 12 months [52], while the study conducted by Wang et al. was a non-blinded RCT comparing the combination of BoNT-A and rehabilitation training with rehabilitation training alone among 48 children aged between 8 and 18 months [53]. Both studies demonstrated that BoNT treatment was associated with a significant decrease in the incidence of spasticity and a considerable improvement in movement range.

In recent years, the role of BoNT-A in reducing the pain caused by CP-induced spasticity has been increasingly evaluated, and a recently published pooled analysis of data from three phase III studies documented that IncoBoNT-A could significantly reduce spasticity-related pain in children and adolescents with CP [54].

Given its favorable safety profile, the use of BoNT-A in pediatric patients with CP is well-known and established [55,56]. Most adverse events, including death, have been observed in children diagnosed with CP who received off-label, high doses of BoNT for the off-label treatment of muscle spasticity [57]. Adverse reactions are generally mild-to-moderate and local, such as muscle weakness, lethargy, dysphagia, asthenia, and fatigue. Systemic adverse reactions like urinary incontinence and dysphagia are less common, and they are potentially related to the distant spread of the toxin after injection [58]. In February 2024, Facciorusso et al. published a bibliometric analysis of research concerning BoNT-A treatment of spasticity in both adult and pediatric patients. In this paper, the authors exhaustively described the available evidence concerning the use BoNT in children, confirming its effectiveness and highlighting safety concerns regarding muscle atrophy, muscle strength reduction, and a loss of contractile elements associated with BoNT-A use [59].

To date, only OnaBoNT-A and AboBoNT-A are approved for the treatment of CP in pediatric patients. Although all the above-mentioned RCTs proved the efficacy and safety of BoNT-A, evidence coming these studies is generally of low certainty due to several limitations [60]. Most of them had a small sample size and, as such, a low statistical power, making it difficult to draw firm conclusions on BoNT efficacy. Several RCTs had a partially blinded or no blinded study design [32,33], and some of them used fixed doses and injections on fixed muscles, thus not being generalizable to every type of CP [38,48].

### 2.2. Dystonia

Dystonia is a movement disorder characterized by involuntary continuous or recurrent muscle contractions, resulting in abnormal and repetitive movements or postures [61]. These kinds of movements could be patterned, twisting, and tremulous, are triggered or worsened by voluntary action, and are frequently present in individuals with CP [62,63].

Dystonia arises from a brain network disorder involving different brain regions, such as basal ganglia, thalamus, sensorimotor cortex, and cerebellum [64]. This disorder is characterized by three main pathological aspects, i.e., loss of inhibition, sensory abnormalities, and maladaptive neuroplasticity [65,66,67].

Along with physical support, oral medications (e.g., levodopa, anticholinergic drugs, baclofen, tetrabenazine), and neurosurgical procedures, BoNT is one of the available therapeutic options for childhood dystonia. In particular, OnaBoNT-A, AboBoNT-A, IncoBoNT-A, and RimaBoNT-B are FDA-approved for cervical dystonia in adults, and only OnaBoNT-A is approved for individuals aged 12 years and older with blepharospasm. However, off-label use is common for the treatment of other dystonias, such as focal laryngeal, limb and oromandibular dystonia [68,69], although evidence from large and well-designed RCTs is currently lacking.

The rationale for using BoNT for the treatment of dystonia is represented by its ability to temporarily inhibit the release of acetylcholine from the presynaptic terminal of motor nerves innervating the muscle fibers, thus preventing the depolarization of the post-synaptic membrane and ultimately resulting in muscle relaxation [70,71].

Beyond its direct action, several factors can alter BoNT effectiveness and safety, including the physical diffusion of the molecule from the injection site and its potential migration to distant areas through either axonal transport (movement along nerve fibers) or hematogenous transport (movement via the bloodstream) [72].

As such, dosing is a critical aspect of BoNT therapy, and it is mainly determined by the number of target muscles and their corresponding BoNT doses, which indicate the extent of their involvement in dystonia [73].

Evidence concerning the effectiveness and safety of BoNT for the treatment of pediatric patients affected by dystonia is still sparce. Most clinical studies mainly involved adult patients with specific dystonic phenotypes, such as cervical dystonia. Treatment guidelines are currently based on knowledge from studies conducted with adults and on the experience of medical professionals [73].

Based on successful treatments of adults with dystonic disorders [74], the efficacy of BoNT-B was evaluated in an open label study conducted on 29 children with spastic or dystonic movement disorders for a total of 62 treatment sessions [75]. In over half of these treatments, an improvement of the motor function was achieved, while no clinical effect was observed in less than 10% of the patients [75].

Such evidence was further confirmed by a small open-label trial conducted on 7 children aged between 2 and 15 year affected by CP and upper extremity dystonia, which showed that BoNT-B significantly improved the speed of outward reaching in children with arm dystonia [76].

Side effects related to BoNT-A for the treatment of dystonia may vary depending on the area affected by the disease, and they are generally mild and transient [77]. Diplopia, dry eye, and ptosis are the most common adverse events when it is used for the treatment of blepharospasm [78,79], and choking and mild breathiness are the most common adverse events when it is used for spasmodic dystonia [80].

### 2.3. Strabismus

Strabismus is an ocular misalignment characterized by the turning of one eye, which can occur intermittently or persistently. This condition affects up to 5% of the general population and may reach up to 50% among specific populations, such as individuals with CP [81,82].

BoNT has emerged as an alternative to surgery for a variety of subtypes of strabismus, but its use in pediatric patients is not as well studied as in adults, mainly due to the perceived challenges of administering it to children, including the need for sedation and potential complications arising from BoNT leakage into the eye levator palpebrae superioris muscle, thus resulting in ptosis [83]. One of the advantages of BoNT injections compared to surgical treatment is the reduced duration of general anesthesia, which is particularly important given the FDA’s warning that prolonged anesthesia for children under 3 years of age can affect brain development [84].

To date, 3 RCTs evaluating the efficacy of BoNT for the treatment of children with strabismus compared with standard surgery have been published, with controversial findings [85,86,87]. The first two RCTs date back to 1998 and 1999 and enrolled 47 children with acquired esotropia and 55 children with infantile esotropia, respectively. Patients were randomly assigned to two different treatment approaches: either reoperation or the administration of BoNT. The authors of the trials compared these two groups in terms of the percentage of successful motor outcomes (defined as ≤8 prism diopters) and the percentage change in deviation and concluded that BoNT injections could be as effective as reoperation in children with strabismus [86,87]. Conversely, in 2021, an unblinded RCT conducted on 101 children with large-angle infantile esotropia (≥40 prism diopters) showed that surgery was more effective than BoNT in achieving complete response (i.e., orthophoria or residual esotropia of ≤10 prism diopters). However, BoNT injection was confirmed to be a safe and effective alternative in children aged 24 months or younger and with lesser degrees of esotropia (i.e., ≤60 prism diopters) [85]. Such evidence was corroborated by a previous systematic review and meta-analysis of nine non-randomized trials evaluating the efficacy of BoNT in children with infantile esotropia, published in 2018. This systematic review showed that the overall success rate of BoNT injection into medial recti muscles was 76% (95% confidence interval [CI]: 61–89%), thus representing an effective alternative to strabismus surgery in congenital esotropia, particularly in children with moderate deviations [88].

Controversial evidence is also coming from observational studies. In 2017, Wan et al. conducted a retrospective study using Boston Children’s Hospital billing records and demonstrated the noninferiority of BoNT as compared to standard strabismus surgery in children with acute onset esotropia [89]. In more detail, 16 children treated with BoNT injections were compared to 33 children undergoing surgery, and no significant difference in the success rate (i.e., the achievement of a final horizontal deviation of 10 prism diopters or less) between the two groups was observed at either 6 months or 18 months [89].

On the contrary, a recently published retrospective observational study examining the medical records of 246 patients under the age of 6 years diagnosed with infantile esotropia showed that surgical intervention was more effective in achieving orthotropia and a deviation of up to 10 prism diopters than BoNT-A injection, particularly in children with angles >30 prism diopters. Such results suggest that BoNT could be used as an alternative to surgery only in cases of small-to-moderate angle deviations (<30 prism diopters) [90].

BoNT is generally well-tolerated for the treatment of strabismus, with the main disadvantages over surgery including a prolonged time of misalignment and a possible ptosis after injection, which can last up to 3 months [85,86,87,88].

In conclusion, BoNT chemodenervation seems to be less effective than standard strabismus surgery in children with long-standing esotropia and those with large-angle esotropia. However, the evidence concerning the use of BoNT for the treatment of strabismus in children is of low certainty and controversial, thus making it difficult to draw robust conclusions whether BoNT may be an alternative to strabismus surgery [91]. Future RCTs should ensure rigorous design and thorough analysis of outcome data to establish high-certainty evidence.

### 2.4. Pediatric Sialorrhea

Sialorrhea, also known as drooling or hypersalivation, is the involuntary discharge of saliva or other contents from the oral cavity due to a lack of coordination of the orofacial and neck muscles. This condition is prevalent among numerous patients and can significantly affect their health and overall quality of life [92]. While drooling is considered normal in children under the age of 4, it becomes problematic as they grow older [93,94]. The mechanisms responsible for sialorrhea can be attributed to hypertrophied salivary glands, heightened saliva production, and an incomplete swallowing mechanism resulting from a lack of neuromuscular control of the oral muscles [95,96].

BoNT is an effective treatment for sialorrhea due to its ability to block acetylcholine release from the cholinergic nerve terminals to the salivary glands [97]. Its effectiveness has been widely demonstrated in adult patients affected by neurodegenerative disorders such as Parkinson’s disease and amyotrophic lateral sclerosis [98,99,100,101], as well as in children. To date, IncoBoNT-A is the only BoNT formulation to be approved by the FDA for the symptomatic treatment of chronic sialorrhea due to neurological or neurodevelopmental disorders in children and adolescents aged 2 to 17 years and weighing ≥12 kg. Local anesthesia, sedation, or anesthesia in combination with sedation may be administered to children and adolescents prior to BoNT injection, after a careful benefit-risk evaluation [102]. As BoNT has not been studied in children weighing less than 12 kg, no dosing recommendations can be made for these patients.

Since 2021, six systematic reviews concerning the management of sialorrhea in pediatric patients have been published, with a total of 60 studies included (29 observational studies, 26 interventional studies, and 5 case series) [103,104,105,106,107,108]. Four of these systematic reviews evaluated the efficacy and safety of BoNT for the treatment of sialorrhea due to any etiology [103,104,105,106], and two for the treatment of sialorrhea due to neurological disorders [108] and CP specifically [107]. The findings of these systematic reviews demonstrated that BoNT is effective and safe in relieving sialorrhea symptoms. When compared to surgical treatments, BoNT was found to be less effective in reducing the severity of drool, but with an overall lower risk of adverse effects [106,108].

The side effects mostly reported in children receiving BoNT for the treatment of sialorrhea include dry mouth, pain and swelling, and dysphagia, which is likely due to toxin diffusion into adjacent musculature and soft tissues leading to subsequent muscle weakness [109,110,111,112]. In this regard, it has been demonstrated that ultrasound-guided injection is a safe procedure, ensuring the accuracy of injection, providing correct positioning of the needle injection within the salivary gland and decreasing iatrogenic damage to adjacent structures by reducing adverse effects [113,114,115,116].

A worldwide consensus is lacking regarding the primary assessment tool to quantify drooling and the follow-up duration. Both subjective and objective metrics have been employed to evaluate adverse effects, severity, and clinical benefits during treatment [103].

### 2.5. Pediatric Neurogenic Detrusor Overactivity

NDO is a bladder dysfunction causing involuntary detrusor contractions during bladder filling, leading to an increased risk of pressure transmission to the upper urinary tract and/or significant incontinence [117,118]. This condition is frequently observed in patients with neurological diseases such as multiple sclerosis and spinal cord injury [119]. Intravesical OnaBoNT-A is currently the only formulation approved by the FDA in 2020 for intravesical use in children older than 5 years with NDO and inadequate response or intolerance to anticholinergic drugs [120]. The introduction of this drug for use in children offered a less-invasive and safe option for the management of this condition by avoiding major reconstructive surgery when first-line interventions, including intermittent catheterization and anticholinergic drugs, fail [118]. BoNT-A can potentially both reduce the risk of renal deterioration by creating a lower pressure urinary reservoir and improve quality of life by improving bladder continence. As such, it is likely that the use of BoNT-A will be integrated into clinical practice guidelines in the near future [118]. Nevertheless, definitive outcomes in pediatric settings remain elusive.

Clinical studies assessing the effectiveness of BoNT-A intravesical injections in children have produced promising results, leading to improved urodynamic outcomes (i.e., increased bladder capacity and reduced bladder pressure) [121,122,123,124]. A systematic review of both interventional and observational studies demonstrated that BoNT-A injection was safe and effective for the treatment of medically unresponsive neurogenic bladder in children, although patient satisfaction with the procedure was controversial [124].

Such findings have been further confirmed by a recent 48-week phase III RCT that compared three doses of OnaBoNT-A (i.e., 50, 100, and 200 U) for the treatment of NDO in children [125]. The study included 113 children, aged from 5 to 17 years, with NDO and urinary incontinence, defined as at least four episodes of daytime urinary incontinence documented in a two-day bladder diary. Results of this study showed that BoNT-A intravesical injections led to significantly increased urine volume and maximum cystometric capacity, as well as to a decreased maximum detrusor pressure during the storage phase, especially with higher BoNT-A doses (200 U vs. 50 U). Patients enrolled in this RCT were enrolled in a repeat-treatment extension trial, in which they could either receive blinded dose escalations or continue with their current dose, based on their previous treatment response as determined by their provider. A total of 95 patients were enrolled in this extension study and received at least one retreatment. Among the patients who received the 200 U dose, a consistent improvement in daytime urinary incontinence episodes was observed from baseline, with more than 75% reporting a positive response on the treatment benefit scale [126].

The main issue of the use of BoNT for NDO treatment is its limited ability to provide absolute symptom control in all patients and the need for repeated administrations to maintain efficacy [126]. Concerning the adverse events occurring during NDO treatment with BoNT, the most commonly reported in the literature were mainly limited to the administration site, including localized pain, tenderness and/or bruising associated with the injection, hematuria, and urinary tract infections [124]. There are currently no known cases of respiratory failure or death following intravesical injection of BoNT-A in children [124].

### 2.6. Congenital Muscular Torticollis

Congenital muscular torticollis (CMT) is a neck deformity characterized by the shortening of the sternocleidomastoid muscle (SCM), with estimated incidence rates ranging from 0.3% to 1.9% of newborns [127]. Infants with CMT generally receive conservative treatment, including massage and neck muscle training; however, if the contracture of the SCM persists beyond one year of age, surgery becomes necessary to prevent craniofacial deformities [128,129]. Although surgery can yield positive results, it may lead to scarring, neck injury, and aesthetic issues due to the postoperative collapse of the SCM on the affected side [130]. In 2005, the first successful treatment of CMT with BoNT was documented, which was able to improve neck rotation and head tilt [131]. Subsequently, the 2018 Guidelines developed by the American Physical Therapy Association (APTA) and the Academy of Pediatric Physical Therapy (APPT) recommended considering BoNT injections for infants with CMT or asymmetric CMT to resolve asymmetry and prevent further deformity when conservative treatment fails [128].

The effectiveness and safety of BoNT for the treatment of CMT was evaluated in a systematic review and meta-analysis including 10 studies (9 cases or case series and 1 non-randomized controlled trial) for a total of 411 patients [132]. The results of this meta-analysis showed that BoNT treatment led to increased degrees of change in range of motion and head tilt, leading to an overall 84% efficacy rate when combined with conservative treatment. However, such conclusions were based only on a small number of patients, highlighting the lack of detailed and homogeneous data regarding the number and frequency of repeated BoNT injections in patients with CMT. Furthermore, the reported number of injections varied widely across the studies, with reports of single, double, and multiple injections [133,134]. Overall, evidence generated so far suggests that BoNT injections for patients with CMT are effective, safe, and can be used in combination with conservative treatments to reduce SCM mass size and improve fibrosis and SCM contracture.

Adverse events reported by children receiving BoNT for the treatment of CMT mainly included bruising, neck pain, transient dysphagia, neck weakness, erythema at the injection site, and fever of unknown origin [132].

## 3. Discussion

The use of BoNT in children represents a promising therapeutic avenue for the treatment of various neurological conditions, including CP, spasticity, dystonia, and other movement disorders. Overall, it was proven to be well tolerated by pediatric patients, with the most commonly reported adverse events following treatment being typically transient, mild, and self-limiting [29,135,136]. This may be due to the fact that only a small quantity of toxin is expected to enter the systemic circulation. BoNT-related adverse events can be broadly categorized into focal (e.g., muscle weakness or soreness, weakness in hand grip, finger drop, and muscle cramps), systemic (e.g., respiratory symptoms and infections, asthenia, flu-like symptoms, dysphagia), and procedural adverse events (e.g., ecchymosis, pain, skin dysesthesia, and rash at the injection site) [137]. While serious side effects like widespread weakness, difficulty swallowing, and breathing problems are uncommon, some patients may frequently experience bleeding at the injection site, muscle weakness potentially affecting movement, local irritation, or a burning sensation. Systemic action of the toxin can occur infrequently, potentially resulting in severe and life-threatening side effects resembling botulism-like symptoms [137]. For this reason, in 2009, the FDA forced manufacturers to put on their product a black box warning due to the potential risk of symptoms of botulism related to systemic spread of BoNT after injections [57]. Furthermore, although long-term use carries minimal risks, there is a small chance of developing resistance to the treatment over time, mainly due to the stimulation of neutralizing antibody formation [138].

Concerning BoNT efficacy, although a large number of clinical studies suggest that different BoNT formulations are generally effective in both approved and off-label pediatric indications, the evidence available is generally debated and of low certainty (Table 1).

Most of the available studies had short follow-up and small sample sizes with low statistical power, thus making it difficult to draw definitive conclusions whether BoNT could be used as a first-line therapy for the treatment of neurological conditions. It should also be noted that many of the RCTs evaluating the efficacy of BoNT in children did not include a placebo control group due to ethical concerns.

Furthermore, many knowledge gaps still exist, including BoNT long-term safety, the impact on the quality of life of both children and their patients/caregivers, and the optimal dosing regimens. In addition, head-to-head comparisons of different BoNT formulations are needed to determine whether one serotype is more effective and safer than another, as well as to establish dosing equivalency and relative antigenicity among different serotypes. Finally, there is limited evidence available regarding the synergistic effects of adjunct therapies (e.g., physiotherapy and occupational therapy) and multimodal treatments in conjunction with BoNT. Research efforts should prioritize addressing these knowledge gaps to optimize therapeutic outcomes and ensure safe and effective use across a broad spectrum of clinical applications. Future studies should be designed with the aim of generating high-certainty evidence to answer all these unmet clinical questions, taking into account the various methodological and ethical issues hindering the conduct of RCTs in children [139,140]. Particularly for off-label conditions, such evidence is also important to develop treatment guidelines defining the appropriate formulation, dosing, and localization for the use of BoNT.

**Figure 1:**
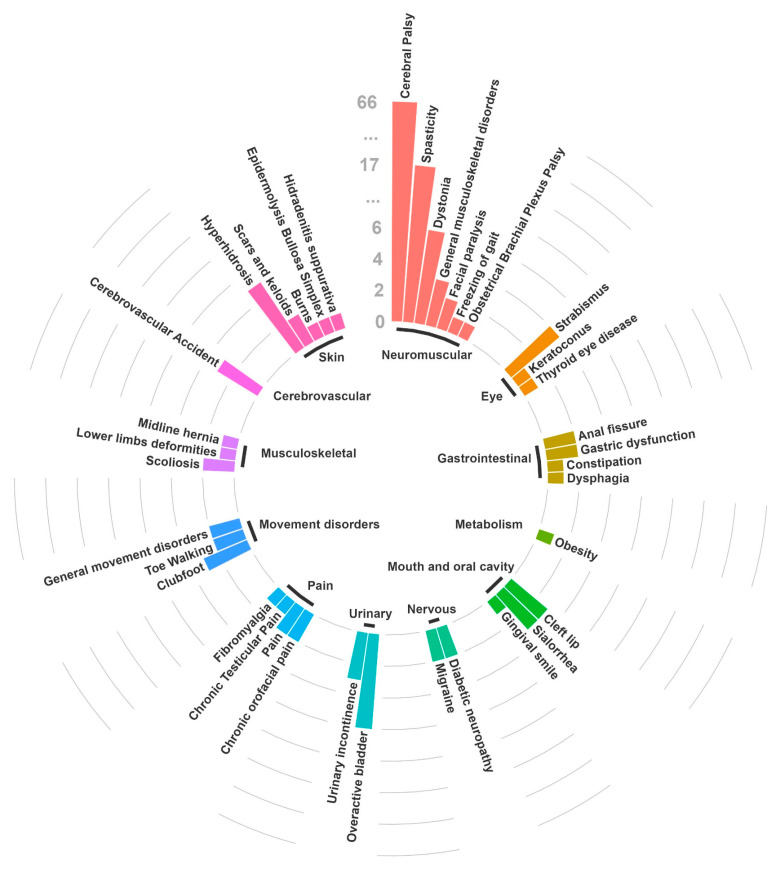
Proportion of studies on BoNT registered in clinicaltrials.gov as of 5 April 2024, stratified by indication of use.

As of 5 April 2024, a total of 154 studies (118 interventional and 36 observational) on the use of different BoNT formulations in children were identified in clinicaltrials.gov (Table S1, Supplementary Materials). In particular, most of the identified studies have been completed (N = 105; 68.2%), 25 (16.2%) are currently ongoing, and for 24 (15.6%) the status was registered as “unknown”. The majority of these studies (N = 65) concerned the use of BoNT for the treatment of CP, followed by other spasticity disorders (N = 14), movement disorders (N = 7), dystonia (N = 6), and pain (N = 6) (Figure 1 and Table S1, Supplementary Material). Some of the other investigated pediatric indications of use included the treatment of dysphagia, obesity, migraine, scoliosis, scars and keloids, keratoconus, hidradenitis suppurativa, epidermolysis bullosa simplex, chronic idiopathic constipation, and chronic anal fissure. These studies have generally small sample sizes, with numbers of enrolled patients ranging from a minimum of 2 to a maximum of 2000.

In addition, novel indication areas for BoNT in neuropathic pain and the related affective disorders and depression are being investigated in ongoing research studies concerning both adult and pediatric patients [141]. Significant advancements have also been achieved in developing non-toxic BoNT fragments for vaccine design for the prevention of botulism, mainly thanks to recombinant DNA technology [142]. In the future, further engineering of BoNT could provide a novel approach with which to better characterize and improve the benefit–risk profile of these neurotoxins. In this regard, stem-cell-based models are a valuable and sensitive system with which to study the biological impacts of BoNT in a cost-effective manner [143].

## Figures and Tables

**Table 1:** Evidence, main study limitations, and knowledge gaps concerning the use of botulinum toxin for different therapeutic indications in children.

Therapeutic Indication	Available Evidence	Main Study Limitations	Knowledge Gaps
Cerebral palsy	BoNT-A was proven to be effective and safe for the treatment of cerebral palsy [30,31,32,33,34,35,36,37,38,40,41,42,43,44,45,46,47,48,49,50]. The therapeutic effects of BoNT-A typically last 3–4 months, thus necessitating repeated treatments [26,27]. BoNT-A dosage depends on various factors such as muscle volume, level of spasticity, and extent of muscle involvement in the pathological pattern [14,28]. Adverse reactions are generally mild-to-moderate and local. Systemic adverse reactions are less common and potentially related to the distant spread of the toxin [57,58,137].	Most studies had a small sample size and a low statistical power [30,31,32,33,34,35,36,37,38,39]. Several RCTs had a partially blinded or no blinded study design [32,33]. Some RCTs did not adequately select the study outcomes or used fixed doses and injections on fixed muscles, thus not being generalizable to every type of cerebral palsy [38,48].	Long-term sustainability of BoNT-A effects. Long-term safety of BoNT-A. The combined effect of BoNT-A and other therapies (e.g., occupational therapy or physiotherapy) on cerebral palsy-related spasticity is still uncertain. Lack of evidence on the use of BoNT-A in children aged <2 years is lacking. Head-to-head comparison between different BoNT-formulation is lacking.
Dystonia	BoNT was proved to be effective for achieving improvement of the motor function and the speed of outward reaching in children with arm dystonia [76]. Side effects may vary depending on the area affected by the disease, and they are generally mild and transient [77,78,79,80].	Evidence in children is still sparce, as most clinical studies mainly involved adult patients with specific dystonic phenotypes.	Lack of robust evidence in children, particularly in those aged <12 years.
Strabismus	BoNT chemodenervation is generally less effective than surgery in children with long-standing esotropia and those with large-angle esotropia, while it could be a valid alternative in children with small to moderate angle deviations [88]. The main disadvantages over surgery include a prolonged time of misalignment and a possible ptosis after injection [85,86,87,88].	Evidence coming from both RCTs and observational studies is controversial [85,86,87,89,90]. Most RCTs had a small sample size and a low statistical power [85,86,87].	It is still uncertain whether BoNT could serve as an independent treatment option for certain types of strabismus, potentially replacing the need for surgery. Head-to-head comparison between different BoNT formulation is lacking.
Sialorrhea	BoNT is effective and safe in relieving sialorrhea symptoms in children [103,104,105,106,107,108]. BoNT was found to be less effective than surgery in reducing the severity of drool, but with an overall lower risk of adverse effects [106,108]. Side effects mostly include dry mouth, pain and swelling, dysphagia, probably due to toxin diffusion into adjacent musculature and soft tissues leading to subsequent muscle weakness [109,110,111,112].	Lack of worldwide consensus concerning the primary assessment tool to quantify drooling, and the follow-up duration.	Head-to-head comparison between different BoNT formulation is lacking.
Neurogenic detrusor overactivity	BoNT-A injection is safe and effective for the treatment of neurogenic bladder in children, leading to consistent improvement in daytime urinary incontinence episodes [121,122,123,124,125,126]. Side effects following BoNT injection mainly include localized pain, tenderness and/or bruising associated with the injection, hematuria, and urinary tract infections [124,126].	Lack of placebo-controlled trials and with long-term follow-up periods.	Head-to-head comparison between different BoNT formulation is lacking.
Congenital muscular torticollis	BoNT treatment led to improvements in range of motion and head tilt, especially when combined with conservative treatment [132]. Adverse events most commonly reported included bruising, neck pain, transient dysphagia, neck weakness, erythema at the injection site, and fever of unknown origin [132].	Evidence comes from studies with a small number of patients [132]. Lack of data regarding the number and frequency of repeated BoNT injections.	Lack of RCTs evaluating BoNT safety in children. Lack of consensus concerning the number of injections needed. Need of longitudinal studies to ascertain the effects of referral and intervention timing on body structure and functional outcomes.

Abbreviations: BoNT = botulinum toxin; RCTs = randomized controlled trials. This holds true specifically concerning pediatric CP as a recently published study evaluating the quality of the available systematic reviews of interventions for children affected by CP demonstrated that the confidence level for almost 90% of them was critically low [60].

## Data Availability

No new data were created or analyzed in this study. Data sharing is not applicable to this article.

## Supplementary Materials

The following supporting information can be downloaded at https://www.mdpi.com/article/10.3390/toxins16070306/s1: Table S1: Interventional and observational studies related to botulinum toxin registered on clinicaltrials.gov as of 5 April 2024.
