# Supplementary material for: Understanding Clinical Effectiveness and Safety Implications of Botulinum Toxin in Children: A Narrative Review of the Literature
Source: Toxins (Basel). 2024 Jul 4;16(7):306. doi: 10.3390/toxins16070306 (PMC11281390; doi:10.3390/toxins16070306)
Supplement: Supplementary file 1 [file toxins-16-00306-s001.zip › toxins-3012839-supplementary.pdf]

Understanding Clinical Effectiveness and Safety Implications of Botulinum Toxin in Children: A Narrative Review of the Literature

Salvatore Crisafulli, Francesco Ciccimarra, Zakir Khan, Francesco Maccarrone and Gianluca Trifirò

Table S1. Interventional and observational studies related to botulinum toxin registered on clinicaltrials.gov as of April 5, 2024.

NCT Number	Status	Condition	Age of study population	Phase	Patients enrolled
**Interventional studies**					
NCT04166175	Completed	Anal fissure	16 years	N.A.	96
NCT02964728	Completed	Burning Mouth Syndrome	All ages	II	6
NCT06174155	Ongoing	Burns	6 - 18 years	N.A.	60
NCT02400619	Completed	Cerebral palsy	5 - 55 years	I	70
NCT00261131	Completed	Cerebral palsy	3 - 18 years	III	250
NCT05126693	Completed	Cerebral palsy	4 - 11 years	N.A.	45
NCT01603615	Completed	Cerebral palsy	2 - 17 years	III	220
NCT01603641	Completed	Cerebral palsy	2 - 17 years	III	370
NCT01603628	Completed	Cerebral palsy	2 - 16 years	III	384
NCT01787344	Completed	Cerebral palsy	2 - 10 years	III	144
NCT01893411	Completed	Cerebral palsy	2 - 17 years	III	311
NCT01905683	Completed	Cerebral palsy	2 - 17 years	III	370
NCT04554238	Completed	Cerebral palsy	6 - 8 years	N.A.	23
NCT02002884	Completed	Cerebral palsy	2 - 17 years	III	351
NCT02188277	Completed	Cerebral palsy	2 - 12 years	II	64
NCT05294874	Completed	Cerebral palsy	5 - 10 years	N.A.	45
NCT01437644	Completed	Cerebral palsy	2 - 15 years	III	54
NCT01377883	Completed	Cerebral palsy	2 - 18 years	N.A.	25
NCT01251380	Completed	Cerebral palsy	2 - 17 years	III	216
NCT01249417	Completed	Cerebral palsy	2 - 17 years	III	241
NCT02546999	Completed	Cerebral palsy	4 - 17 years	IV	61
NCT00060957	Completed	Cerebral palsy	3 - 12 years	II	40
NCT05312125	Completed	Cerebral palsy	5 - 10 years	N.A.	19
NCT04179682	Completed	Cerebral palsy	3 - 6 years	N.A.	6
NCT03704155	Completed	Cerebral palsy	5 - 12 years	N.A.	24
NCT01276015	Completed	Cerebral palsy	25 months - 9 years	IV	18
NCT00133861	Completed	Cerebral palsy	7 - 17 years	II/III	23
NCT03680196	Completed	Cerebral palsy	2 - 10 years	N.A.	20
NCT03302871	Completed	Cerebral palsy	5 - 16 years	III	40
NCT02853240	Completed	Cerebral palsy	8 - 17 years	N.A.	20
NCT00467207	Completed	Cerebral palsy	10 - 17 years	II	10
NCT02596412	Completed	Cerebral palsy	3 - 8 years	N.A.	80
NCT03580174	Completed	Cerebral palsy	3 - 12 years	N.A.	24
NCT02917967	Completed	Cerebral palsy	2 - 18 years	N.A.	31

NCT03306212	Completed	Cerebral palsy	3 - 18 years	III	34
NCT03472261	Completed	Cerebral palsy	2 - 18 years	III	35
NCT03469999	Completed	Cerebral palsy	5 - 17 years	III	12
NCT04328168	Completed	Cerebral palsy	7 - 10 years	N.A.	28
NCT00178217	Completed	Cerebral palsy	1 - 17 years	I/II	101
NCT00238641	Completed	Cerebral palsy	2 - 18 years	I/II	10
NCT00552721	Completed	Cerebral palsy	7 - 18 years	N.A.	14
NCT03553446	Completed	Cerebral palsy	3 - 12 years	N.A.	23
NCT05724030	Ongoing	Cerebral palsy	4 - 16 years	N.A.	75
NCT06128616	Ongoing	Cerebral palsy	3 - 17 years	III	40
NCT05627921	Ongoing	Cerebral palsy	3 - 18 years	N.A.	40
NCT03616067	Ongoing	Cerebral palsy	4 - 18 years	III	130
NCT06218316	Ongoing	Cerebral palsy	4 - 4 years	N.A.	75
NCT05340439	Ongoing	Cerebral palsy	2 - 17 years	II	30
NCT04177186	Unknown	Cerebral palsy	5 - 17 years	N.A.	40
NCT04940143	Unknown	Cerebral palsy	5 - 13 years	N.A.	19
NCT00173745	Unknown	Cerebral palsy	6 - 21 years	N.A.	20
NCT02586142	Completed	Cerebrovascular accident	All ages	III	30
NCT02557737	Unknown	Cerebrovascular accident	All ages	III	40
NCT02469948	Unknown	Cerebrovascular accident	All ages	III	40
NCT02058836	Completed	Chronic testicular pain	All ages	II	8
NCT02247193	Completed	Cleft lip	0 - 6 months	I/II	30
NCT05559281	Completed	Cleft lip	0 - 1 year	N.A.	22
NCT00152347	Completed	Clubfoot	1 day - 2 months	N.A.	36
NCT00152334	Completed	Clubfoot	1 day - 2 years	N.A.	500
NCT00474032	Unknown	Clubfoot	0 - 12 years	N.A.	2000
NCT02361749	Unknown	Constipation	2 - 12 years	IV	40
NCT05296759	Completed	Diabetic neuropathy	All ages	IV	30
NCT00845897	Completed	Diabetic neuropathy	All ages	N.A.	17
NCT04695600	Ongoing	Dysphagia	≥ 14 years	II/III	100
NCT05095493	Completed	Dystonia	18 months - 80 years	N.A.	54
NCT02420106	Completed	Dystonia	2 - 100 years	N.A.	10
NCT00936533	Unknown	Epidermolysis bullosa simplex	≥ 16 years	II	40
NCT00989209	Completed	Facial paralysis	16 - 62 years	N.A.	25
NCT02078791	Completed	Fibromyalgia	14 years	IV	66
NCT06094309	Ongoing	Freezing of gait	All ages	N.A.	20
NCT01631604	Unknown	General movement disorders	≥ 5 years	N.A.	N.R.
NCT06207955	Ongoing	General musculoskeletal disorders	16 - 45 years	N.A.	39
NCT04810429	Completed	General musculoskeletal disorders	12 - 60 years	IV	15
NCT03812965	Completed	Gingival smile	All ages	II	20
NCT03103074	Completed	Hidradenitis suppurativa	All ages	N.A.	20
NCT00168415	Completed	Hyperhidrosis	12 - 17 years	IV	144
NCT03203174	Completed	Hyperhidrosis	12 years	I	13
NCT00004480	Completed	Hyperhidrosis	16 years	N.A.	N.R.
NCT01930604	Unknown	Hyperhidrosis	16 years	II	588
NCT00175669	Completed	Idiopathic toe walking	5 - 15 years	II	32
NCT01590693	Completed	Idiopathic toe-walking	5 - 15 years	N.A.	52
NCT01691651	Completed	Keratoconus	10 - 40 years	N.A.	40

NCT00412035	Completed	Lower limbs deformities	5 - 21 years	III	125
NCT04131348	Completed	Midline hernia	All ages	N.A.	80
NCT03055767	Completed	Migraine	8 - 17 years	II	17
NCT01662492	Completed	Migraine	12 - 17 years	III	125
NCT05605886	Ongoing	Myofascial pain syndrome	17 - 50 years	I	24
NCT03079557	Completed	Obesity	12 - 18 years	II	10
NCT03198702	Ongoing	Obstetrical brachial plexus palsy	10 - 11 months	III	62
NCT02816151	Completed	Overactive bladder	3 - 15 years	III	29
NCT01716624	Completed	Overactive bladder	5 - 20 years	III	20
NCT05911594	Completed	Overactive bladder	7 - 16 years	N.A.	128
NCT00175123	Unknown	Overactive bladder	2 - 16 years	IV	30
NCT00320281	Completed	Pain	≥ 15 years	IV	19
NCT00403273	Completed	Pain	All ages	II	54
NCT01429402	Unknown	Scars and keloids	3 months - 45 years	III	120
NCT06230146	Ongoing	Scars and keloids	10 years	I/II	45
NCT04922983	Ongoing	Scoliosis	14 - 18 years	II	42
NCT03935295	Ongoing	Scoliosis	10 - 16 years	IV	90
NCT05097079	Ongoing	Sialorrhea	3 - 17 years	III	108
NCT02270736	Completed	Sialorrhea	2 - 17 years	III	256
NCT01859507	Completed	Spasticity	16 - 44 years	N.A.	32
NCT02106351	Completed	Spasticity	2 - 17 years	III	212
NCT00178646	Completed	Spasticity	≥ 12 years	IV	33
NCT03307135	Completed	Spasticity	All ages	N.A.	67
NCT00819065	Completed	Spasticity	≥ 2 years	III	56
NCT00015795	Completed	Spasticity	All ages	I	30
NCT01256021	Completed	Spasticity	2 - 18 years	IV	212
NCT03521076	Ongoing	Spasticity	4 years	N.A.	49
NCT03585569	Unknown	Spasticity	All ages	III	30
NCT01584843	Completed	Strabismus	≥ 12 years	III	41
NCT03459092	Completed	Strabismus	1 - 17 years	III	63
NCT03266549	Unknown	Strabismus	≥ 6 months	N.A.	46
NCT00370734	Unknown	Thyroid eye disease	All ages	II/III	50
NCT01603602	Completed	Upper limb spasticity	2 - 16 years	III	235
NCT01226706	Completed	Urinary incontinence	17 - 80 years	N.A.	21
NCT01852045	Completed	Urinary incontinence	5 - 17 years	III	114
NCT01852058	Completed	Urinary incontinence	5 - 17 years	III	95
**Observational studies**					
NCT05405634	Ongoing	Anal fissure	≥ 16 years	N.A.	80
NCT03112434	Completed	Cerebral palsy	5 - 18 years	N.A.	16
NCT03888443	Completed	Cerebral palsy	6 - 17 years	N.A.	40
NCT05295563	Completed	Cerebral palsy	5 - 18 years	N.A.	29
NCT03149263	Completed	Cerebral palsy	2 - 18 years	N.A.	80
NCT00503620	Completed	Cerebral palsy	4 - 12 years	N.A.	11
NCT03234413	Completed	Cerebral palsy	2 - 18 years	N.A.	118
NCT03788317	Completed	Cerebral palsy	2 - 18 years	N.A.	29
NCT03344952	Completed	Cerebral palsy	2 - 18 years	N.A.	503
NCT02096549	Completed	Cerebral palsy	3 - 18 years	N.A.	20
NCT05013619	Completed	Cerebral palsy	8 - 18 years	N.A.	75

NCT04792606	Ongoing	Cerebral palsy	2 - 19 years	N.A.	1000
NCT05497609	Ongoing	Cerebral palsy	2 - 18 years	N.A.	50
NCT05691556	Ongoing	Cerebral palsy	5 - 18 years	N.A.	20
NCT05207124	Ongoing	Cerebral palsy	5 - 18 years	N.A.	60
NCT04290689	Unknown	Cerebral palsy	4 - 16 years	N.A.	20
NCT01987882	Unknown	Cerebral palsy	2 - 18 years	N.A.	500
NCT01335100	Unknown	Cerebral palsy	5 - 18 years	N.A.	10
NCT03047499	Unknown	Cleft lip	3 - 6 months	N.A.	2
NCT05103202	Completed	Dystonia	All ages	N.A.	105
NCT02175693	Completed	Dystonia	All ages	N.A.	1647
NCT02175719	Completed	Dystonia	All ages	N.A.	150
NCT00535938	Completed	Dystonia, spasticity, hyperhidrosis, cerebral palsy	≥ 14 years	N.A.	1372
NCT04250844	Unknown	Gastric dysfunction	30 days - 18 years	N.A.	100
NCT03904004	Unknown	Gastric dysfunction	All ages	N.A.	60
NCT00001208	Ongoing	General movement disorders	2 years	N.A.	2000
NCT01655862	Completed	General musculoskeletal disorders	16 years	N.A.	62
NCT03407183	Unknown	Overactive bladder	All ages	N.A.	20
NCT03888001	Unknown	Overactive bladder	6 years	N.A.	20
NCT06101160	Completed	Sialorrhea	4 - 12 years	N.A.	61
NCT03384927	Ongoing	Spasticity	All ages	N.A.	96
NCT04207632	Unknown	Spasticity	5 - 99 years	N.A.	5
NCT01390922	Completed	Spasticity	All ages	N.A.	1038
NCT03017729	Completed	Spasticity	2 - 17 years	N.A.	242
NCT00746304	Completed	Strabismus	1 day - 5 years	N.A.	164
NCT06150729	Ongoing	Spasticity	2 - 17 years	N.A.	106

Abbreviations: N.A. = not applicable; N.R. = not reported.
